# Effect of Environmental and Spatial Factors on Multi‐Diversity in Mt. Huangshan

**DOI:** 10.1002/ece3.71841

**Published:** 2025-08-13

**Authors:** Ting Lv, Rong Zhao, Ningjie Wang, Lei Xie, Shuifei Chen, Hui Ding, Yanming Fang

**Affiliations:** ^1^ Ankang University Ankang China; ^2^ College of Life Sciences Nanjing Forestry University Nanjing China; ^3^ Research Center for Nature Conservation and Biodiversity, State Environmental Protection Scientific Observation and Research Station for Ecology and Environment of Wuyi Mountains, State Environmental Protection Key Laboratory on Biosafety Nanjing Institute of Environmental Sciences, Ministry of Ecology and Environment Nanjing China

**Keywords:** community assembly, ecological niche, environmental factors, functional diversity, phylogenetic diversity, species diversity

## Abstract

The plant species in Huangshan Mountain (Mt. Huangshan) are abundant. Nevertheless, a comprehensive analysis of the relationship between biodiversity and environmental factors is lacking. This study aimed to analyze how multiple factors affect biodiversity and explain the mechanisms underlying community assembly processes and species maintenance. Thus, we compared the different biodiversities among Evergreen Broadleaf Forest (EBF), Deciduous Broadleaf Forest (DBF), and Mixed Needleleaf and Broadleaf Forest (MNBF) in a total of 75 plots in each community using a non‐parametric Wilcoxon rank‐sum test. Then, we employed Mantel tests to quantify the influence of ecological conditions, including spatial predictors, topographical variables, soil composition, and climate factors, on the three forest communities. Our findings revealed the following: (1) The species diversity and phylogenetic diversity within the EBF and MNBF were found to be higher than that in the DBF. The functional richness (FRic) in the MNBF was higher compared to the other two communities, whereas the functional evenness (FEiv), functional divergence (FDiv), and functional dispersion (FDis) in the DBF were the highest. (2) The species diversity of different canopy plants was positively correlated with phylogenetic diversity and functional richness, yet negatively correlated with FEve, FDis, and FDiv. Phylogenetic diversity exhibited a negative correlation with functional diversity for both total and shrub layers, whereas it showed a positive correlation with functional diversity for the tree layer. The type of forest canopy exhibited the strongest correlation with functional diversity. (3) In the redundancy analysis, environmental factors had a stronger influence on biodiversity than spatial distance, indicating that deterministic processes had a greater impact than random processes. The findings underscore the importance of key factors that are often overlooked in the work of protecting Mt. Huangshan, such as elevation, aspect, total phosphorus (TP), and precipitation of the driest month (Bio.14) etc. This provides theoretical guidance for the ecological restoration of forest vegetation in Mt. Huangshan.

## Graphical abstract

This study compared and analyzed the differences and relationships between species diversity, phylogenetic diversity, and functional diversity, and highlighted the roles of spatial distance and environmental variables in three different forests across Huangshan Mountain Resort.

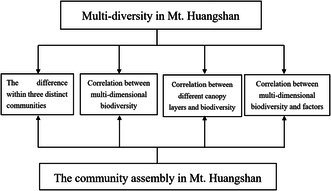

## 1 Introduction

The habitats of species are influenced by the advancement of human society, which in turn affected the biodiversity of communities. Biodiversity pertains to the variety and variability of organisms and their ecological complexes (Albassatneh et al. 2023), encompassing species diversity (SD), phylogenetic diversity (PD), and functional diversity (FD). Functional traits are typically characterized by polygenic inheritance (Kelly et al. 2014; Schulz et al. 2019). Closely related species tend to exhibit more similarity in function compared to distantly related ones. Consequently, communities with a higher level of phylogenetic diversity are likely to possess greater functional diversity (Srivastava et al. 2012; Boris and Georgy 2025). Nevertheless, the relationship between these two aspects is not as straightforward as it seems. This is because trait evolution may not necessarily align with the phylogenetic structure; for instance, trait homology can result from convergence or reversal. Moreover, the traits investigated in temperate and tropical tree communities display relatively weak phylogenetic signals (Swenson 2012). In fact, the question of whether there is synergistic change among the multi‐dimensional biodiversity in forest communities is still inconclusive (Li et al. 2024). A community characterized by high species diversity and phylogenetic diversity might concurrently possess low functional diversity (Purschke et al. 2013; Perronne et al. 2014). As species diversity increases, functional diversity generally rises (Bu et al. 2014). Consequently, the integrated analysis of SD, PD, and FD can uncover the evolutionary history of the community and its specific phenotypic traits. Simultaneously, this approach is conducive to disclosing the ecological processes of evolution and functional assembly (Zhou et al. 2021; Cintra et al. 2024).

Research into the distribution and maintenance mechanisms of biodiversity has long been a prominent topic within community ecology (Portela et al. 2023). Grasping how environmental variables impact biodiversity is crucial information for predicting community assembly processes (Götzenberger et al. 2012; de Bello et al. 2021). Two hypotheses exist regarding the maintenance mechanism of community biodiversity. These are the niche theory (Fernandez‐Going et al. 2013; Mason et al. 2013), which is founded on deterministic processes, and the neutral theory (Soininen et al. 2007; Wang et al. 2025), which is based on stochastic processes. The former emphasizes environmental filtering and competition exclusion as two opposing fundamental drivers of community composition (Webb et al. 2002). Environmental filtration is an abiotic factor that prevents a species from establishing or persisting in a specific location, allowing species with specific characteristics or phenotypes to survive and excluding those that are not adapted to the environment (Kraft et al. 2015). Competitive exclusion highlights the competition between two species with similar ecological niches for similar environmental resources, which gives rise to unstable co‐existence (Mayfield and Levine 2010). However, the neutral theory posits that the random dispersal of ecologically equivalent individuals is pivotal in maintaining community species (Legendre et al. 2009; Rosindell et al. 2012). Phylogenetic diversity represents the evolutionary relationships among species. When ecological niches are preserved within an evolutionary lineage, environment‐driven filtering can favor closely related and ecologically similar species (Baraloto et al. 2012; Gastauer and Meira‐Neto 2013). Species with a more distant phylogenetic relationship coexisting in a community typically exhibit differences in functional traits, thus enabling efficient resource allocation (Leibold and McPeek 2006). Research findings have indicated that environmental filtration and diffusion restriction often have a synergistic effect during community aggregation (Page and Shanker 2018; Chase et al. 2019). Nevertheless, the significance of these two processes may differ contingent upon the scale of the study and the environmental conditions within the realm of research (Myers et al. 2013; Voronin et al. 2024; Boris and Georgy 2025). The difference of forest group type between plots mainly depends on the degree of community succession and the difference of topography in the Central Yunnan Plateau (Yang et al. 2023a; Yang et al. 2023b). The distribution of arbuscular mycorrhizal fungi (AMF) within the Atlantic forest was shaped by both spatial and habitat heterogeneity (Nóbrega et al. 2025). The distribution pattern of shrub plants in the shrub‐grassland of the Inner Mongolia Plateau was influenced by spatial heterogeneity (Liu et al. 2024a; Liu et al. 2024b). Environmental variables and spatial variables affect the interaction between the internal and external environment of the population at different scales, thus affecting the stability of the community (Lundholm 2009; Stein et al. 2014). Thus, a fundamental comprehension of the alterations in biodiversity within plant communities resulting from external environmental factors has assumed greater significance. Notably, when confronted with challenging factors such as climate change and topography, the evolutionary responses of these species may not occur rapidly enough, potentially leading to their replacement by other species (Galván‐Cisneros et al. 2023).

Mt. Huangshan is home to a rich variety of plant species and features a complex community structure. Nevertheless, the community is confronted with severe threats, including climate warming, biological invasion, human interference, and ecological carrying‐capacity issues. As a result, its biodiversity and ecosystem functions have significantly declined (Xie et al. 2025). Moreover, research on the responses of different types of communities in this region to environmental changes remains less than comprehensive. Currently, studies on Mt. Huangshan predominantly concentrate on a single dimension of biodiversity, yet the relationships among multi‐dimensional biodiversity aspects have not been reported. Through a study of the biodiversity disparities among various communities, the crucial aspects and suitable scales for biodiversity conservation and ecosystem restoration can be ascertained (Wang et al. 2025). Based on the community survey, evergreen broad‐leaved forest (EBF), deciduous broad‐leaved forest (DBF), and mixed broad‐leaved forest (MNBF) were compared to explore the differences and correlations of multi‐dimensional biodiversity in order to address the following three questions:

1. How do species diversity, phylogenetic diversity, and functional diversity vary among different community layers across the three communities?
2. What are the relationships among the diverse aspects of biodiversity?
3. to reveal the main driving forces of forest community construction mechanism in Mt. Huangshan and deduce the mechanism underlying community assembly.

Furthermore, it is necessary to tailor distinct protection measures according to different forest types. To preserve species richness within the native habitats, forest management should prioritize areas characterized by high environmental variability. Additionally, creating a high level of local habitat heterogeneity, encompassing factors such as topography, soil nutrients, and climate, is essential for ensuring a high degree of species diversity. Besides, spatial distance and climate change must be comprehensively taken into account (Wang et al. 2025).

## 2 Materials and Methods

### 2.1 Study Area

This study was conducted at Huangshan Mountain Resort (118°01′–118°17′ E, 30°01′–30°18′ N) in Anhui, China, with a total area of 160.6 km^2^. The study area is located at the northern margin of the central subtropical zone, within the evergreen broad‐eaved forest, red‐soil, and yellow‐soil region, and has a subtropical monsoon climate. During the study period, the mean temperature was 7.8°C, and the annual rainfall was 2394.5 mm. Specifically, 85% of the annual rainfall occurred in summer and autumn (Sang et al. 2022). Mt. Huangshan is mainly composed of pantropical distribution and north temperate distribution plants, showing a common temperate and subtropical nature. Within the zonal vegetation of Mt. Huangshan, evergreen broad‐leaved forest, deciduous broad‐leaved forest, and deciduous broad‐leaved mixed forest are distributed from bottom up. Some dominant species, such as *Castanopsis eyrei* and *Pinus hwangshanensis*, are apex communities with different levels of succession (Zhu et al. 2020).

**FIGURE 1:**
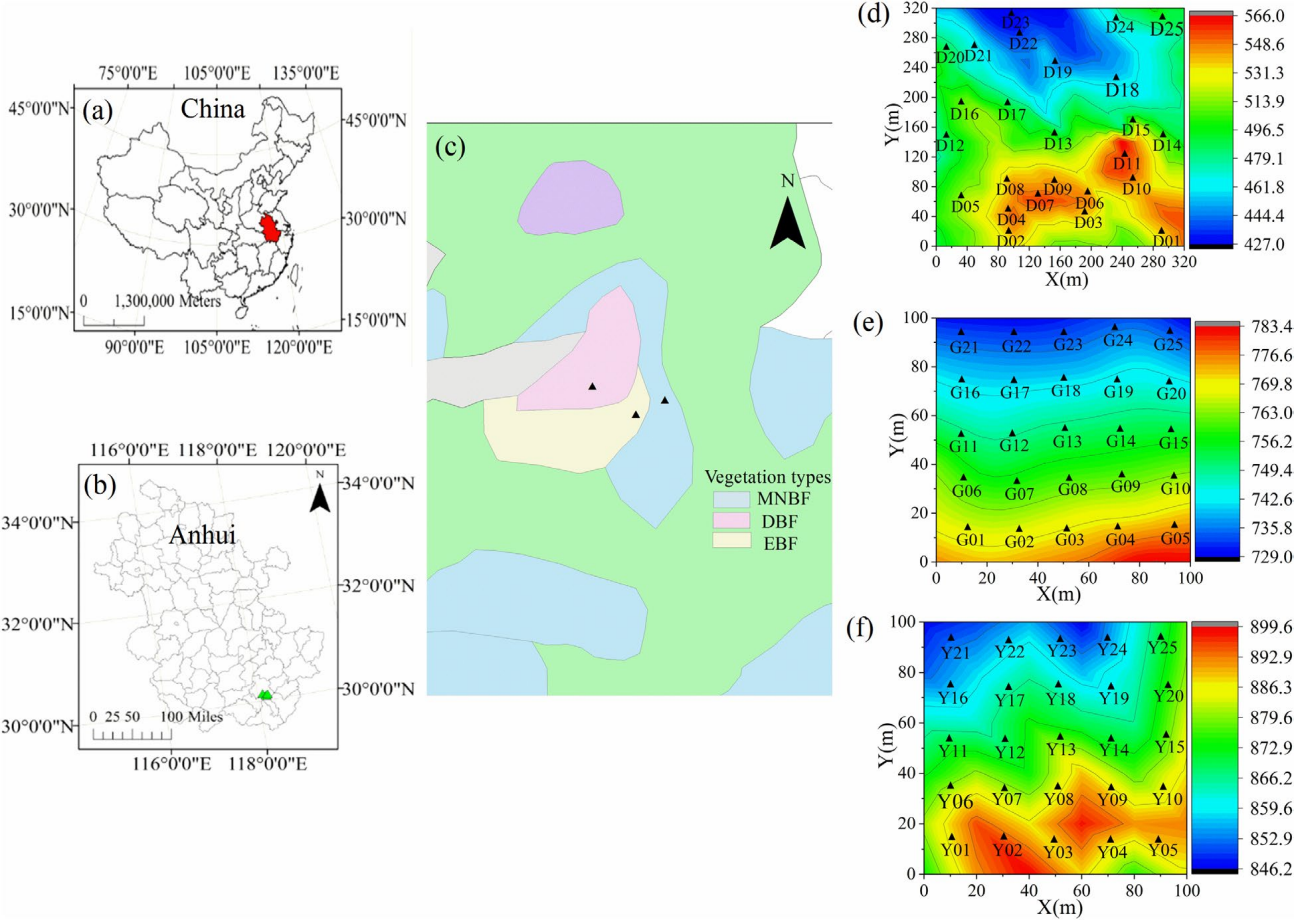
The location (a, b) and vegetation diagram (c) of the three forests situated in Mt. Huangshan, Anhui Province, and China. For each of the forest communities, specifically the Evergreen—Broadleaf Forest (EBF) (d), the Deciduous—Broadleaf Forest (DBF) (e), and the Mixed Needle—leaf and Broad—leaf Forest (MNBF) (f), there are 25 plots.

Building upon previous research, Evergreen Broadleaf Forest (EBF, 118°6′38″ E, 30°8′26″ N) with *C. eyrei* and *Eurya nitida* as the dominant species, Deciduous Broadleaf Forest (DBF, 118°10′7″ E, 30°6′5″ N) with *Cyclobalanopsis myrsinifolia* and *Lindera rubronervia* as the dominant species, Mixed Needleleaf and Broadleaf Forest (MNBF, 118°11′28″ E, 30°6′41″ N) with *E. nitida* and *Rhododendron ovatum* as the dominant species were selected as research sites (Table 1, Figure 1). In each area, a total of 25 plots, each covering an area of 400 m^2^, were surveyed. Subsequently, for the convenience of the survey, each of these plots was further partitioned into 16 sub‐plots, each with an area of 25 m^2^. All sample plots were investigated on‐site in line with the CTFS (Centre for Tropical Forest Science) investigation technical specifications. For all woody plants having a diameter at breast height (DBH) of ≥ 1 cm, their species names, DBH, tree heights, heights under branches, crown widths, coordinates (X, Y), and growth status were identified. Significantly, according to the tree height, each forest community could be categorized into the shrub layer (1 m < *h* < 6 m) and the tree layer (*h* > 6 m).

**TABLE 1:** Plots of three forest communities at Mt. Huangshan.

Forest types	Elevation (m)	Stand density (trees/hm^2^)	Plot area (m^2^)	Plots (number)	Total area (m^2^)
EBF	440–540	3890	400	25	10,000
DBF	600–700	1788	400	25	10,000
MNBF	800–900	5345	400	25	10,000

### 2.2 Selection and Measurement of Leaf

**FIGURE 2:**
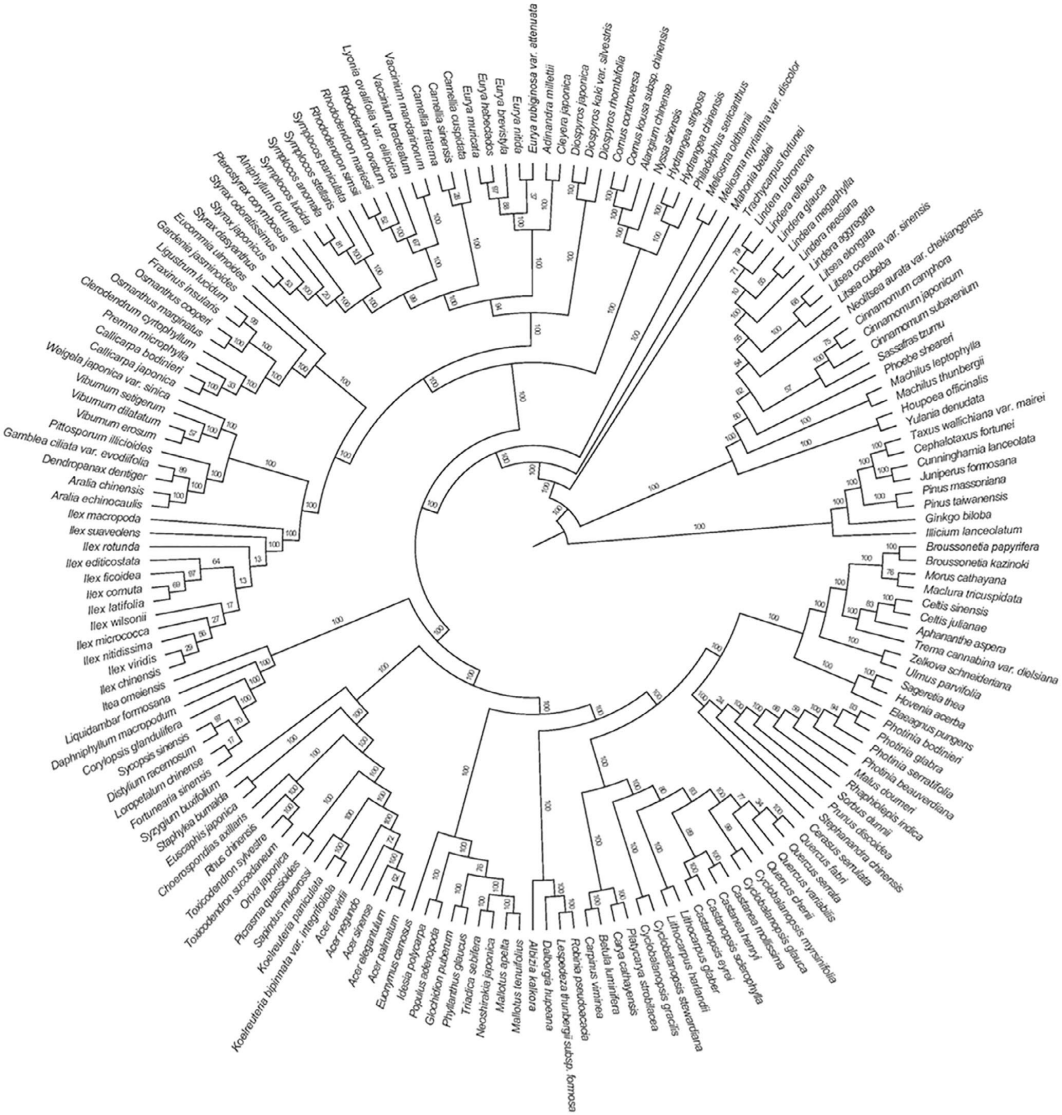
The hypermatrix was constructed using the sequence data of three DNA barcode loci (ITS2 + *rbc*L + *mat*K). Additionally, the constraint tree constructed according to the APG IV system was employed to jointly construct the community phylogenetic tree of 176 plant species. The numbers located at the nodes of the circular tree graph represent the support rate.

112 species were investigated in EBF, 97 species were investigated in DBF, and 86 species were investigated in MNBF, which belong to 55 families and 116 genera. Total DNA was extracted from silica‐dried leaf materials of these species. The leaf materials were healthy, fresh, sterile, and included young infected leaves. A plant DNA extraction kit (Beijing Tiangen Technology Company, Beijing, China, DP305) was used for the extraction. The amplified ITS2, *rbc*L, and *mat*K sequences were sequenced and then a phylogenetic tree was constructed using a constraint tree and DNA supermatrix (Figure 2).

A total of 1024 individuals were obtained from three communities, including 443 individuals in EBF, 297 individuals in DBF, and 284 individuals in MNBF. Leaf traits have attracted special attention because of their sensitivity to climate change and their capacity to reflect the acquisition and utilization of plant resources (Xing et al. 2023). They are capable of mirroring the adaptability of plants to habitats throughout community succession (Ye et al. 2022). Moreover, they are associated with aspects such as species distribution, life‐history strategies, and resource utilization rates (Akram et al. 2022). Based on the main factors affecting plant growth are light, water, and soil nutrients, six leaf traits were selected for exploring functional diversity, including leaf thickness (LT/mm), leaf area (LA/cm^2^), specific leaf area (SLA/cm^2^·g^‐1^), the contents of leaf carbon (LCC/mg·g^−1^), nitrogen (LNC/mg·g^−1^) and phosphorus (LPC/mg·g^−1^), which represent the leaf economic spectrum (Liu et al. 2021). The leaf economic spectrum is also capable of effectively characterizing community and ecosystem features, including productivity and ecosystem services (Flores et al. 2014). These six indicators are able to depict the life‐history strategies and trade‐offs of species from diverse perspectives. Variations in LT drive niche differentiation and exert an impact on community structure and ecosystem functions (Cornwell et al. 2006). LA affected light capture and temperature of leaves (Swenson and Enquist 2009). SLA represents one of the crucial traits that mirror the growth and survival strategies of plant leaves. Moreover, it can indicate the capacity of plants to acquire resources like light (Bär Lamas et al. 2016). LCC serves as a crucial parameter for estimating the carbon‐equestration capacity of forests (Perez‐Harguindeguy et al. 2013). LNC significantly influences the photosynthetic efficiency of plants, while LPC impacts the energy‐conversion capacity and growth regulation (Adler et al. 2014). Consequently, these trait parameters can be utilized to explore the adaptive resource strategies of plant individuals and communities in response to diverse environments.

The LT was measured at a position on the leaf that avoided the main vein, using an electronic Vernier caliper with an accuracy of 0.01 mm. The leaf weight (LW) was measured by means of an electronic balance with an accuracy of 0.001 g. After scanning the leaves, the LA was calculated using Image J v.1.8.0, an image‐analysis software. The SLA was calculated using the equation of SLA = LA/LW. The collected leaves were dried and crushed. The nitrogen content was determined using the Kjeldahl nitrogen‐determination method (Cintra et al. 2024). The carbon content was determined according to the method described by Xing et al. (2023), and the phosphorus content was determined by the molybdenum colorimetric‐resistance method (Wang et al. 2023).

### 2.3 Topographical, Soil, Climate and Spatial Indexes

The environmental factors we measured primarily encompass topography, soil, and climate variables. We utilize elevation, aspect, slope, and convexity to characterize the terrain of the study area. These data were acquired through ArcGIS (Legendre et al. 2009). The soil pH was measured via the potentiometric method. The TN (Total Nitrogen) was determined using the Kjeldahl method, and the TP (Total Phosphorus) was evaluated by means of a spectrograph. The climate data, consisting of 19 bioclimatic factors (Table 2), were retrieved from the World Clim Global Climate Dataset (http://www.worldclim.org) according to the geographical coordinates, with the aid of DIVA‐GIS software.

**TABLE 2:** Description of 19 bioclimatic variables.

Variables	Description
Bio.1	Annual mean temperature
Bio.2	Mean diurnal range (mean of monthly [max temp − min temp])
Bio.3	Isothermality, (Bio‐2/Bio‐7)
Bio.4	Temperature seasonality
Bio.5	Max temperature of warmest month
Bio.6	Min temperature of coldest month
Bio.7	Temperature annual range (Bio.5–Bio.6)
Bio.8	Mean temperature of wettest quarter
Bio.9	Mean temperature of driest quarter
Bio.10	Mean temperature of warmest quarter
Bio.11	Mean temperature of coldest quarter
Bio.12	Annual precipitation
Bio.13	Precipitation of wettest month
Bio.14	Precipitation of driest month
Bio.15	Precipitation seasonality coefficient of variation
Bio.16	Precipitation of wettest quarter
Bio.17	Precipitation of driest quarter
Bio.18	Precipitation of warmest quarter
Bio.19	Precipitation of coldest quarter

To remove the collinearity of environmental factors, *Pearson* correlation analysis was performed. Ten variables (*r* < 0.75) with little retained correlation were screened, and a 75 × 10 environmental factor matrix [elevation, aspect, slope, convexity, pH, TP, TN, Bio.3 (Isothermality), Bio.14 (Precipitation of driest month), Bio.15 (Precipitation seasonality coefficient of variation)] was formed for subsequent analysis. Utilizing the longitude and latitude (geographic coordinates) of the plots, the principal coordinates of neighbor matrices (PCNM) for 22 adjacent matrices were generated via the “*SpaceMaker*” function of the “*vegan*” package in R (Dray et al. 2006).

### 2.4 Calculation of Species, Functional and Phylogenetic Diversity

Species richness, Shannon‐wiener index, Simpson index, Pielou evenness index were used to calculate species diversity.

Species richness:R=S

$$\begin{array}{c} \text{Shannon}‐\text{Wiener index}:H=-\sum_{i=1}^{s}P_{i}\ln P_{i} \end{array}$$

$$\text{Simpson index}:D=1-\sum_{i=1}^{s}P_{i}^{2}$$

Pielou evenness index:J=H/LnS

Where *S* denotes the total number of species, and $P_{i}$ represents the proportion of the number of individuals of the *i*‐th species to the total number of individuals within the community (Yang et al. 2023a; Yang et al. 2023b).

We computed Faith's phylogenetic diversity (PD), defined as the total length of the branches of species on the phylogenetic tree (Veldkornet and Adams 2025), using the “*picante*” package in R.

Four components, functional richness (FRic), functional evenness (FEve), functional divergence (FDiv), and functional dispersion (FDis), are employed to depict functional diversity (de Bello et al. 2021). We computed them with “*FD*” package in R.

### 2.5 Relating Biodiversity to Environmental and Spatial Indexes

To conduct significance tests for the differences in species diversity, phylogenetic diversity, and functional diversity among different canopy layers of the three communities, a non‐parametric Wilcoxon rank‐sum test was utilized. This test was computed using the “*ggpubr*” package in R. Then, we used *Pearson* correlation to understand how each diversity component related to each other in the study area. Upon obtaining the variable matrix of the influencing factors, the *Mantel* test was employed to examine the correlations among the environmental matrix, spatial distance matrix, and biodiversity matrix under 999 permutations. The Pearson correlation coefficient (*r* ^2^) among these matrices was computed to determine their correlations and significance levels. The above‐mentioned analysis was accomplished using the “*vegan*” package in R (Wang et al. 2025). Construct and analyze through the molecular ecological network analysis pipeline in the “*tidyverse*” and “*dplyr*” packages. Plotting was performed using the “*ggplot2*” package in R 4.0.2, and the graphs were performed using Origin v.2019.

## 3 Results

### 3.1 Species Diversity, Functional Diversity and Phylogenetic Diversity Within Three Distinct Communities

**FIGURE 3:**
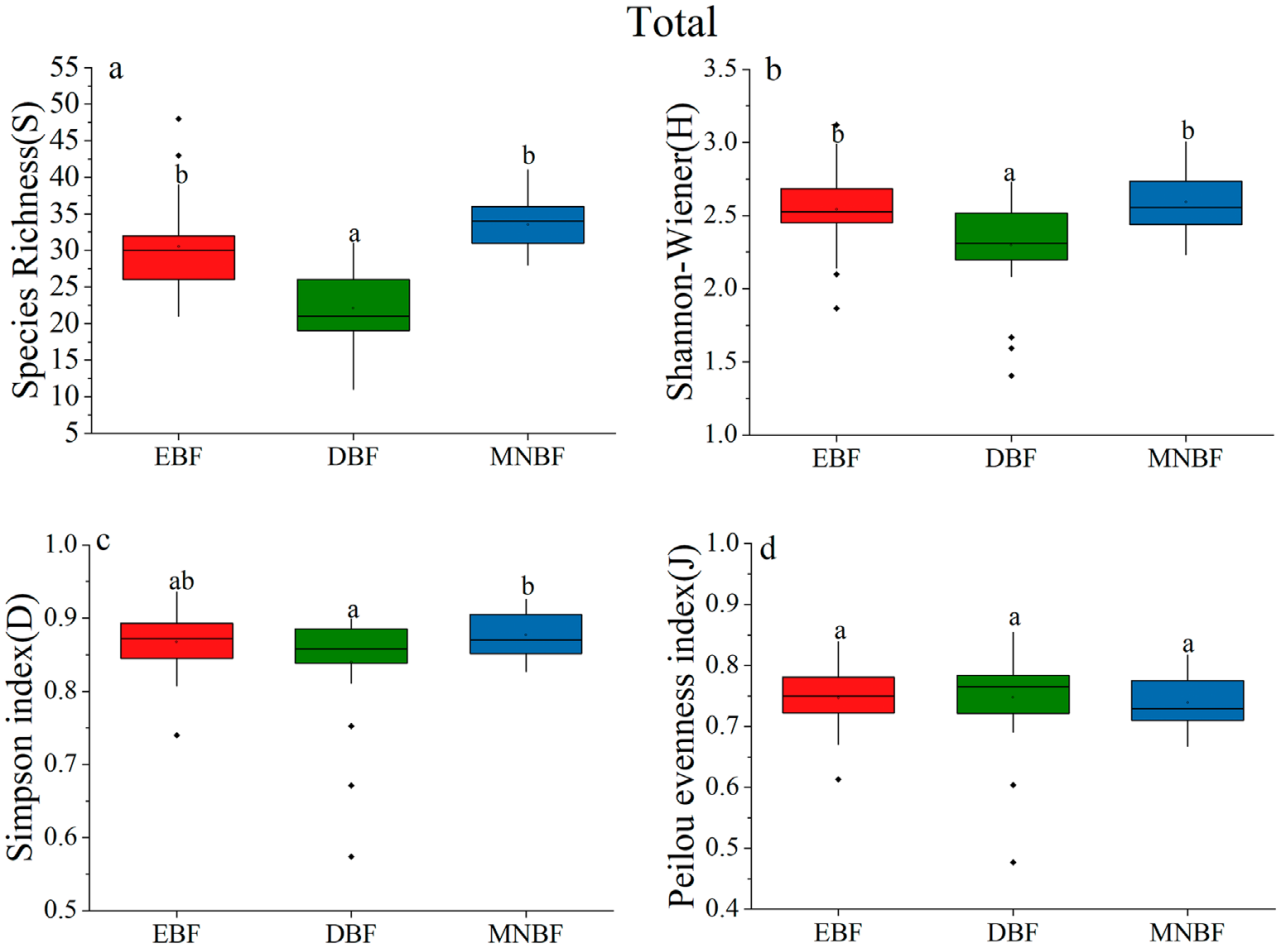
Comparisons of species Richness (a), Shannon–Wiener diversity (b), Simpson index (c), and Pielou evenness index (d) across the total canopy layers of the three forest communities in Mt. Huangshan. Distinct lowercase letters signify that there exist substantial differences among various groups (*p* < 0.05).

**FIGURE 4:**
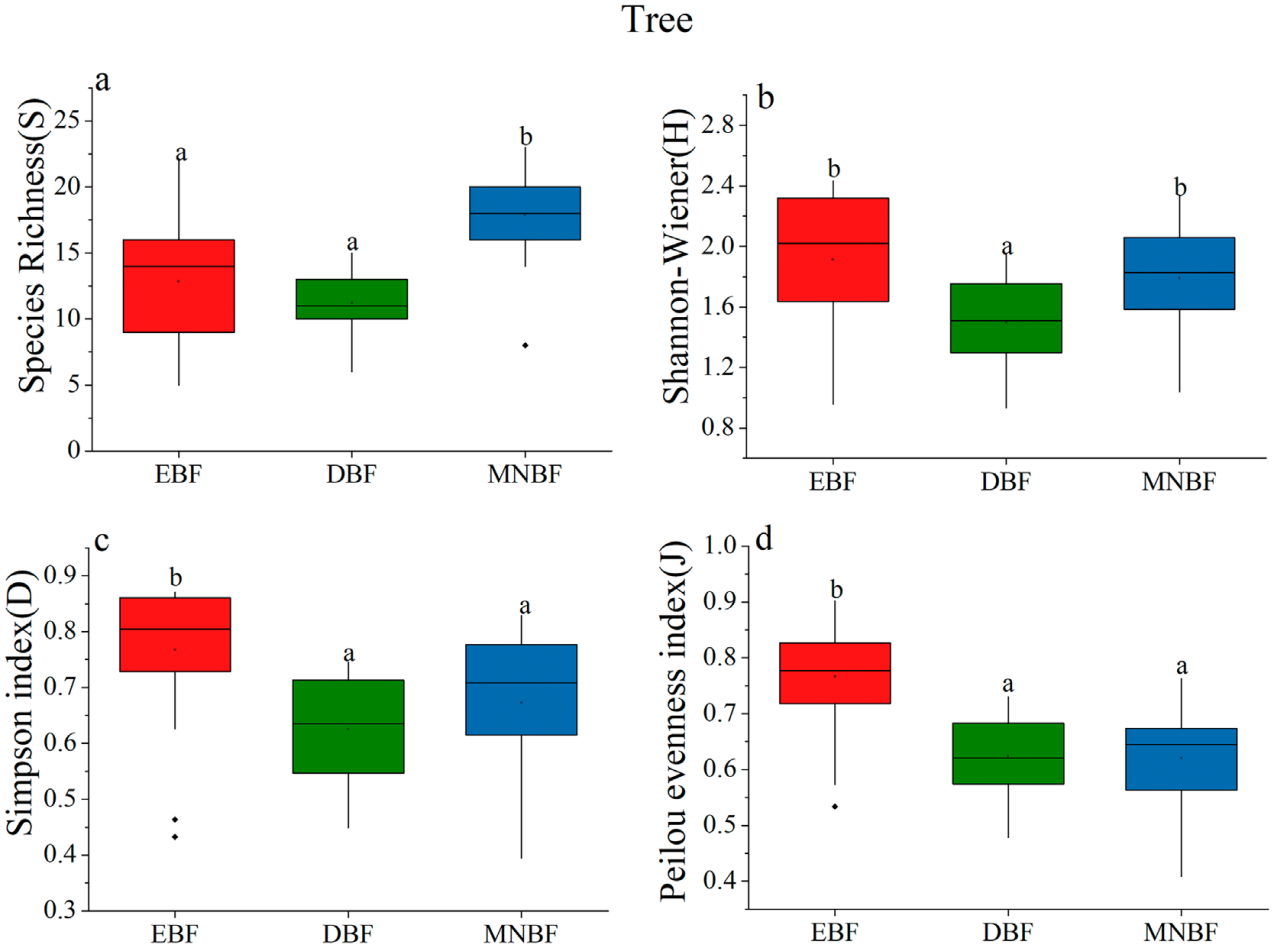
Comparisons of species Richness (a), Shannon–Wiener diversity (b), Simpson index (c), and Pielou evenness index (d) across the tree canopy layers of the three forest communities in Mt. Huangshan. Distinct lowercase letters signify that there exist substantial differences among various groups (*p* < 0.05).

**FIGURE 5:**
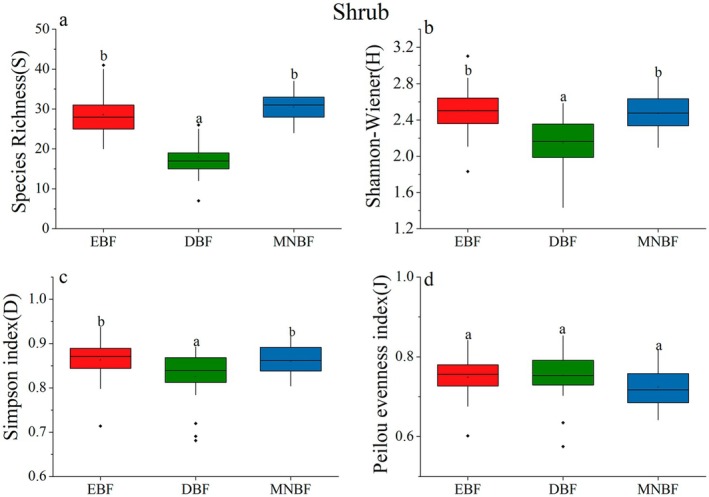
Comparisons of species Richness (a), Shannon–Wiener diversity (b), Simpson index (c), and Pielou evenness index (d) across the shrub canopy layers of the three forest communities in Mt. Huangshan. Distinct lowercase letters signify that there exist substantial differences among various groups (*p* < 0.05).

Significant differences in species diversity were observed among three communities (Table 3, Figures 3, 4, and 5). For the total layer, the species richness (*S*) of EBF and MNBF was significantly higher than that of DBF (Figure 3a), which similar results were found in the shrub layer (Figure 5a). However, *S* of MNBF in the tree layer was significantly higher than that of the other two communities (Figure 4a). The result of Shannon–Wiener followed the order EBF > MNBF > DBF (Figures 3b, 4b, and 5b). The Simpson index indicated that there was the lowest diversity for the tree layer and shrub layer in DBF, and the highest diversity in EBF (Figures 3c, 4c, and 5c). For the Pielou evenness index, there was no significant difference among the three communities in the total layer and shrub layer. In the tree layer, the index of EBF was higher, indicating that the species distribution was more uniform than in the other two communities (Figures 3d, 4d, and 5d).

**TABLE 3:** Species diversity indices for various canopy layers across different forest communities.

Diversity indexes	EBF			DBF			MNBF		
	Total	Tree	Shrub	Total	Tree	Shrub	Total	Tree	Shrub
Species Richness (S)	30.52 ± 6.45	12.84 ± 4.85	28.6 ± 5.41	22.08 ± 5.17	11.2 ± 2.55	17.92 ± 4.78	35.56 ± 3.00	17.88 ± 3.30	30.52 ± 3.33
Shannon–Wiener (H)	2.54 ± 0.27	1.91 ± 0.45	2.50 ± 0.27	2.29 ± 0.33	1.49 ± 0.27	2.14 ± 0.30	2.56 ± 0.19	1.79 ± 0.37	2.47 ± 0.18
Simpson index (D)	0.86 ± 0.04	0.77 ± 0.12	0.86 ± 0.05	0.84 ± 0.07	0.63 ± 0.09	0.82 ± 0.05	0.87 ± 0.03	0.67 ± 0.13	0.85 ± 0.03
Peilou evenness index (J)	0.74 ± 0.05	0.77 ± 0.08	0.75 ± 0.02	0.74 ± 0.07	0.62 ± 0.07	0.75 ± 0.06	0.72 ± 0.04	0.62 ± 0.09	0.72 ± 0.05

**FIGURE 6:**
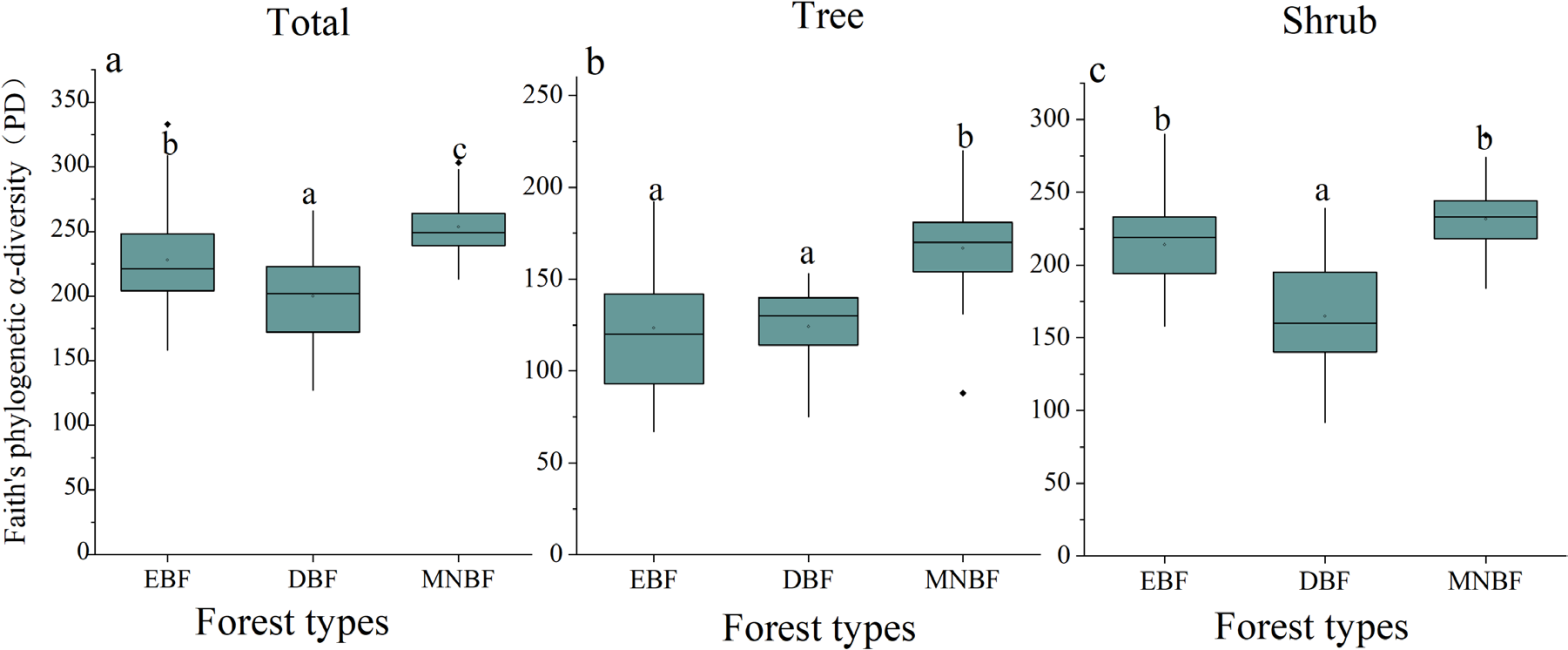
The difference of phylogenetic α‐diversity (PD) between three forest communities for different layers (a, b, c). Distinct lowercase letters signify that there are substantial differences among various groups (*p* < 0.05).

On total layer, the phylogenetic diversity (PD) of the three communities showed a pattern of MNBF > EBF > DBF, and significant differences were observed among them (*p* < 0.001) (Figure 6). Moreover, MNBF still showed the highest phylogenetic diversity in tree and shrub layer. The phylogenetic pattern on shrub layer is consistent with that of total layer, so the phylogenetic diversity of community was mainly determined by shrub layer.

**FIGURE 7:**
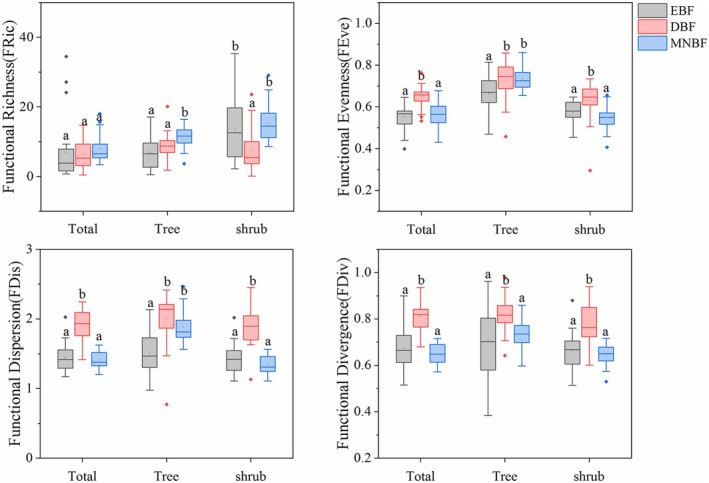
Variations in the functional diversity indices (FRic, FEv, FDis, FDiv) across different canopy layers of the three communities at Mt. Huangshan.

In total layer, there was no significant difference in functional richness (FRic) among the three communities. However, for the tree and shrub layers, the functional richness of MNBF was significantly higher than that of the other two communities (Figure 7). Functional uniformity (FEve), functional dispersion (FDiv) and functional dispersion (FDis) were highest in DBF for the three layers, indicating that this community had high functional diversity, which might be related to the divergence of functional traits (Figure 7).

### 3.2 Correlation Between Multi‐Dimensional Biodiversity in Different Canopy Layers

**FIGURE 8:**
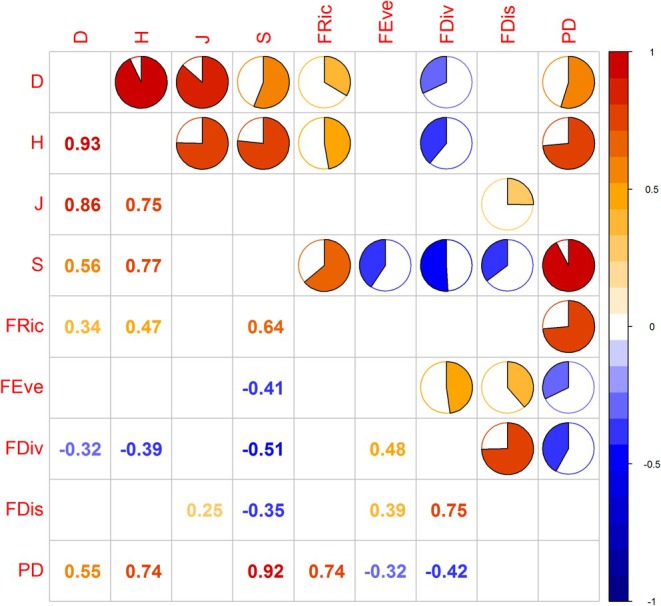
The correlations among the multi‐dimensional biodiversity aspects of the total canopy layer at Mt. Huangshan.

**FIGURE 9:**
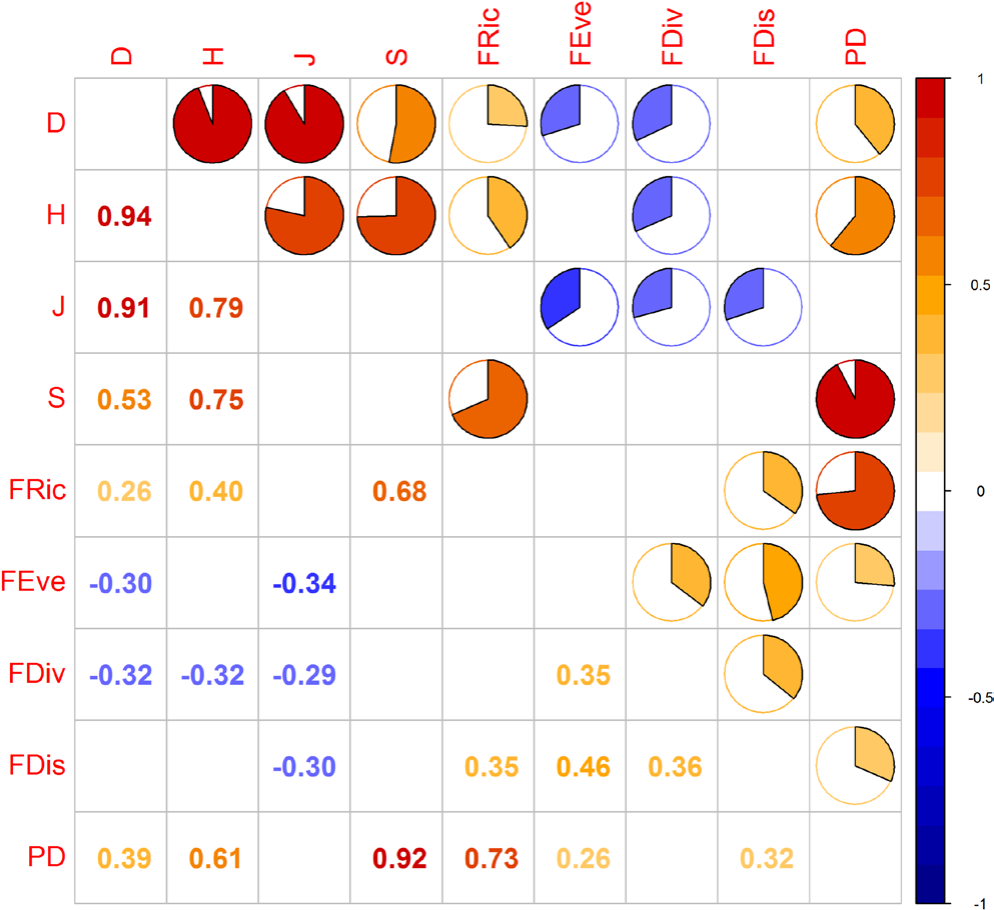
The correlations among the multi‐dimensional biodiversity aspects of the tree canopy layer at Mt. Huangshan.

**FIGURE 10:**
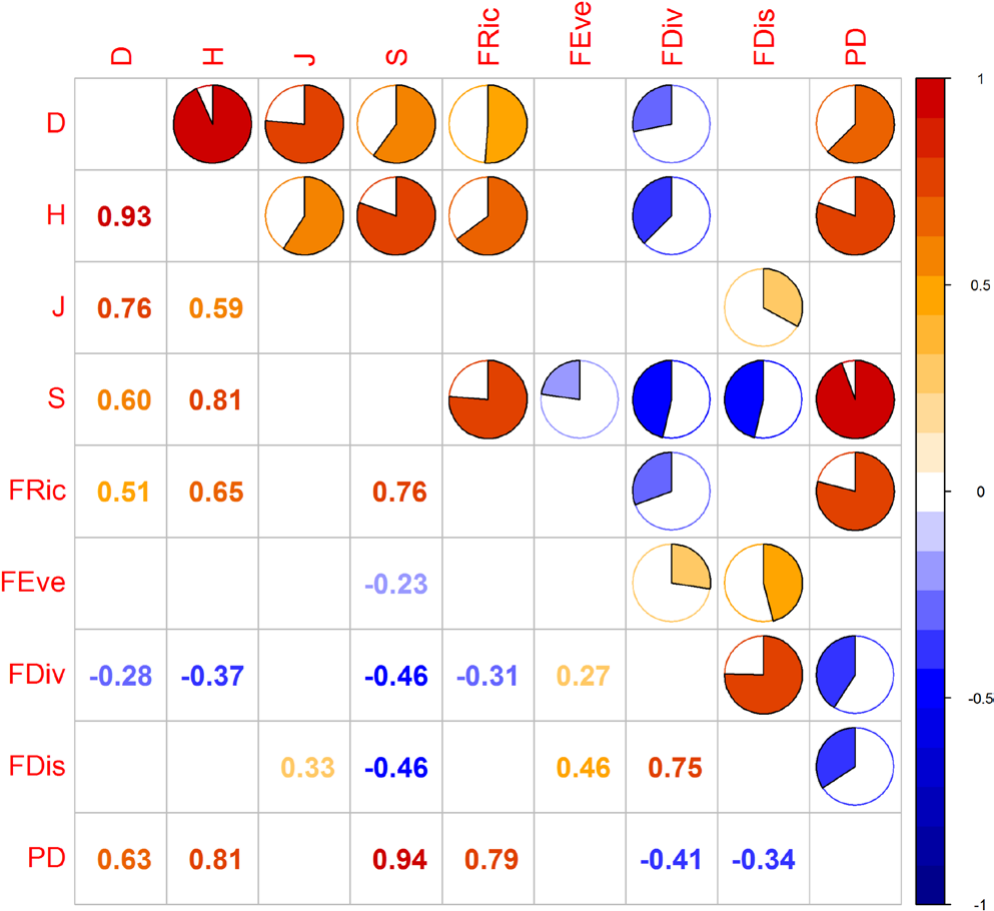
The correlations among the multi‐dimensional biodiversity aspects of the shrub canopy layer at Mt. Huangshan.

For three layers, PD was positively correlated with metrics such as the Simpson index (*D*), Shannon–Wiener index (*H*), and species richness (*S*), yet it was not correlated with the Pielou evenness index (*J*) (Figures 8, 9 and 10). PD was significantly positively correlated with functional richness (FRic). Conversely, it was negatively correlated with functional divergence (FDiv), functional evenness (FEev), and functional dispersion (FDis). FRic and FDiv were significantly positively correlated with species diversity. FEev in the total and shrub layers was negatively correlated with species richness. In the tree layer, it was negatively correlated with both *D* and *J*. FDis was negatively correlated with species richness, yet positively correlated with the Pielou evenness index (*J*).

In addition, there was a certain correlation between the indicators representing the diversity of different dimensions. The four indexes of species diversity showed a significant positive correlation. In the three different canopy layers, the four indicators of functional diversity showed different correlations. FDis for the total layer was significantly positively correlated with FEev and FDiv, while it was significantly negatively correlated with FRic for the shrub layer.

### 3.3 Correlation Between Different Canopy Layers and Biodiversity

**FIGURE 11:**
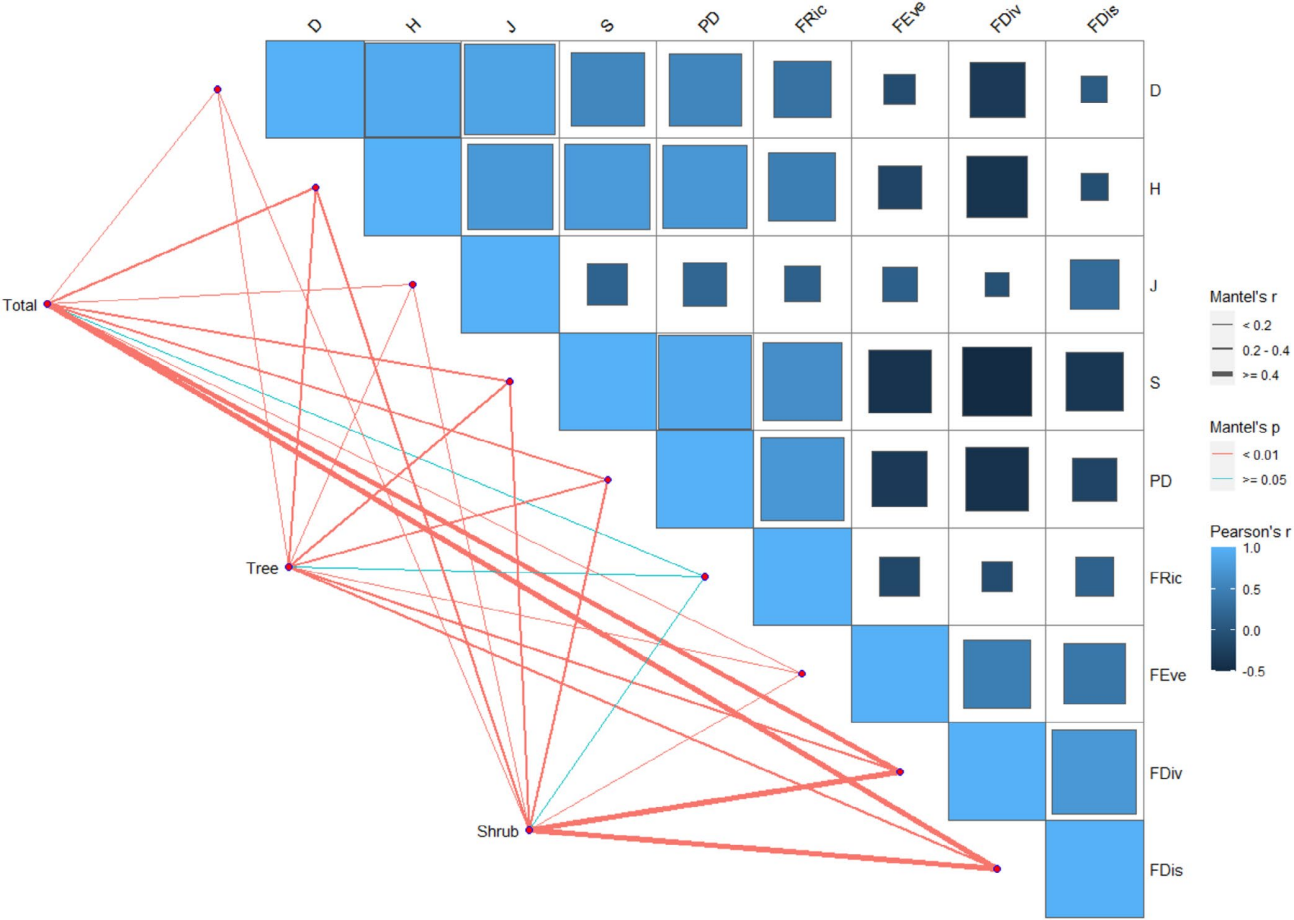
Different canopy layers: driver of species, phylogenetic, and functional diversity. (Pairwise comparisons of biodiversity were presented, where a color gradient indicated Spearman's correlation coefficients. The width of the line corresponds to Mantel's *r* statistic, employed for the respective distance correlations. The color of the line signifies the statistical significance derived from 9999 permutations.)

According to the heat map of Spearman's among different canopy layers (Figure 11), forest type was significantly correlated with functional diversity, followed by phylogenetic diversity, and less significantly correlated with Simpson index and Pielou evenness index in the total plant layer. In the tree layer, the forest type was significantly correlated with both species diversity and phylogenetic diversity, yet it was not correlated with functional richness. In the shrub layer, community type was significantly correlated with FDiv and FDis, but not significantly correlated with FRic, species diversity and phylogenetic diversity. In total, the biodiversity of Mt. Huangshan was dominated by functional diversity, followed by phylogenetic diversity, and species diversity was the lowest, among which Shannon–Wiener index and species richness contributed more to the biodiversity.

### 3.4 Correlation Between Multi‐Dimensional Biodiversity, Environmental Factors and Spatial Variables

**FIGURE 12:**
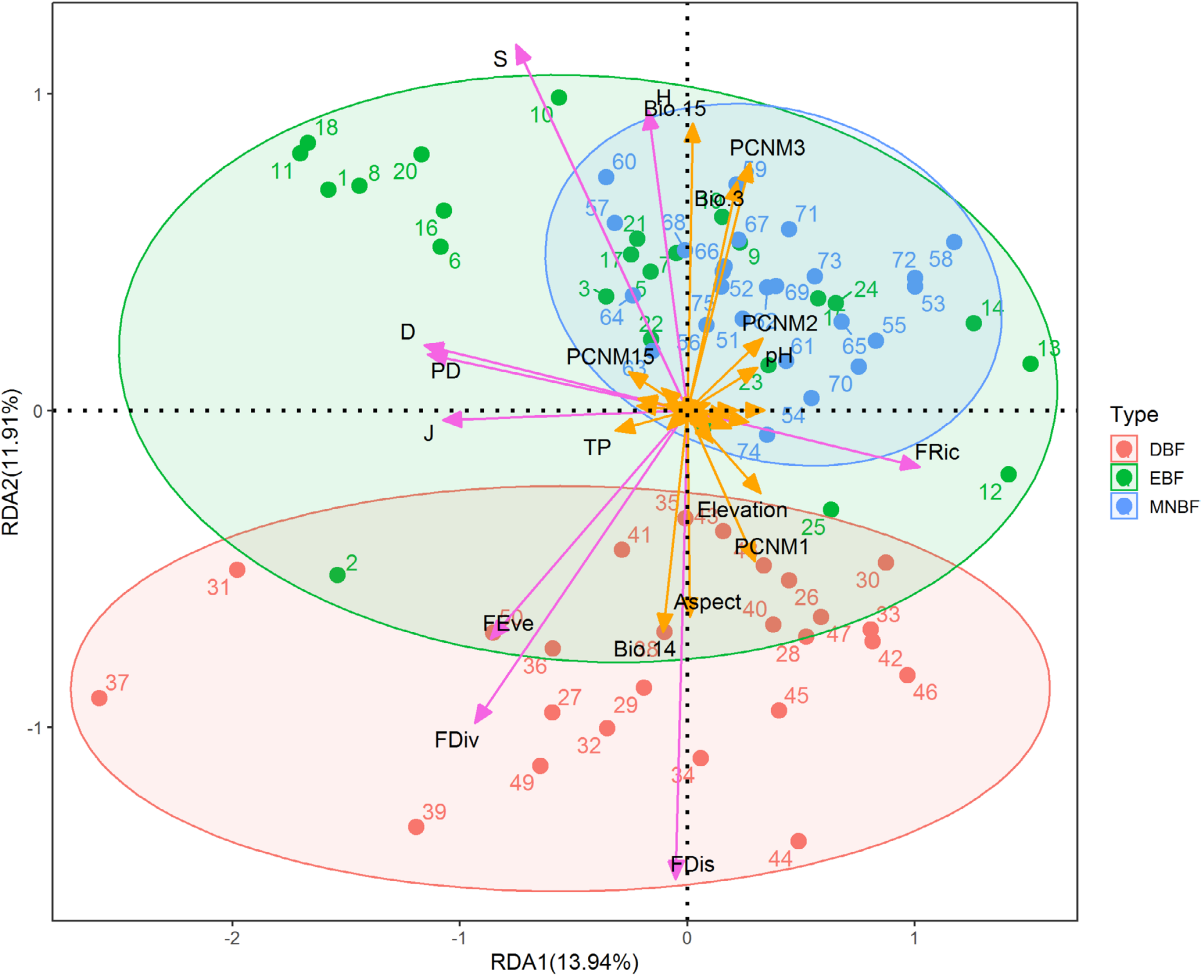
Redundancy analysis (RDA) of the relationships among biodiversity, spatial variables, and environmental factors within the total canopy at Mt. Huangshan.

In total plant layer, the distribution of biodiversity in each plot in MNBF was concentrated, while relatively larger in EBF and DBF (Figure 12), and the overall distribution was skewed to the right on the RDA axis. The results of the correlation analysis indicated that Bio.15, Bio.14, Bio.3, elevation, aspect, pH, and total phosphorus (TP) were the primary environmental factors influencing the biodiversity of Mt. Huangshan. Additionally, the spatial distance was mainly represented by PCNM3, PCNM1, PCNM2, and PCNM15. The correlations between community biodiversity and environmental factors were more robust than those between community biodiversity and spatial distance. In the redundancy analysis, the first two axes of the RDA accounted for 13.94% and 11.91% of the total variance in the data, respectively.

**FIGURE 13:**
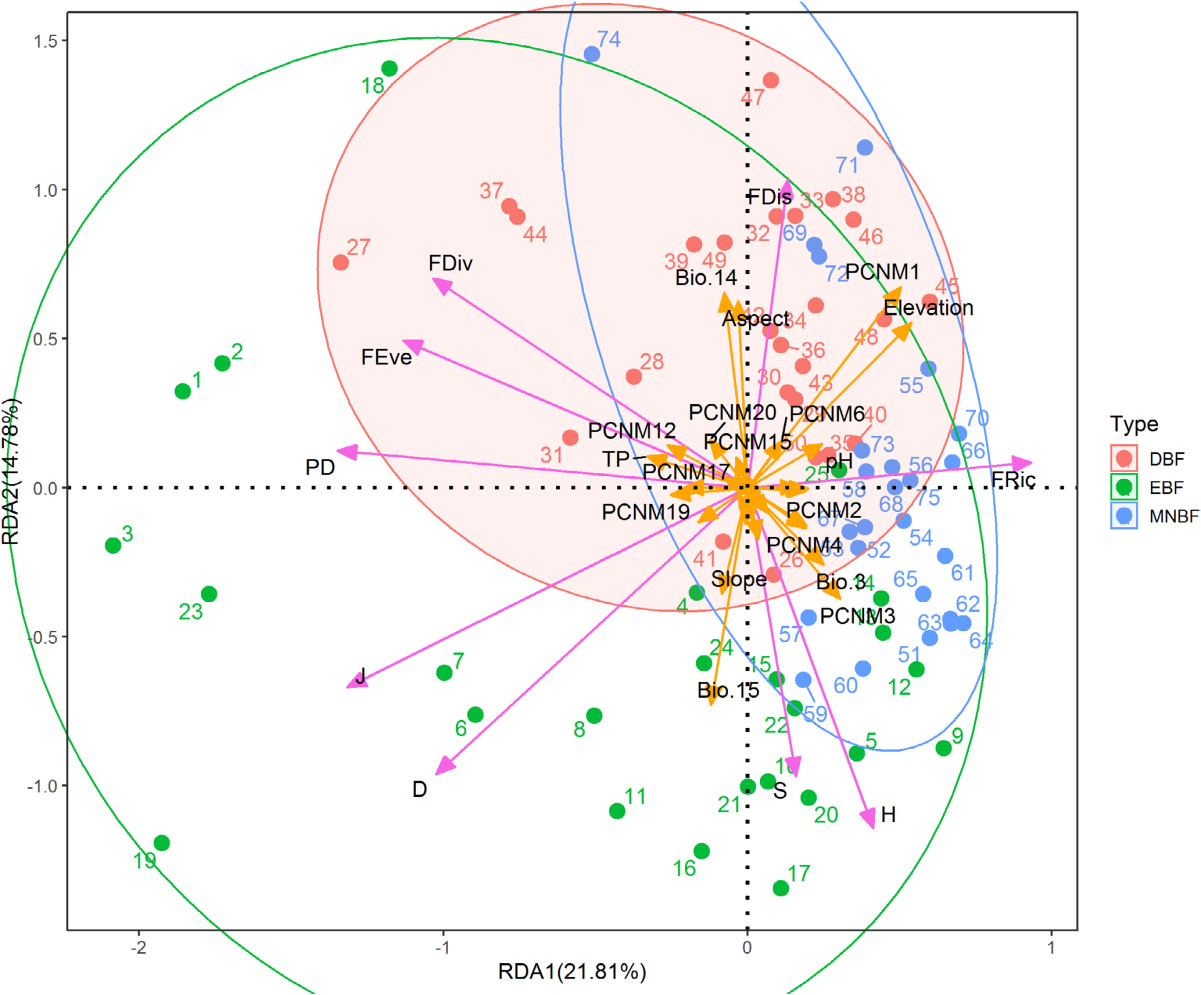
Redundancy Analysis (RDA) of the relationships among biodiversity, spatial variables, and environmental factors within the tree canopy at Mt. Huangshan.

In tree layer, there were significant differences in biodiversity between plots in each community and a high degree of overlap among the three communities, mainly concentrated on the right‐hand side of the RDA axis (Figure 13). According to the RDA ordination diagram, it can be inferred that environmental factors (elevation, aspect, Bio.15, Bio.14, etc.) have a greater influence on the community diversity than spatial distance (PCNM1, PCNM3). In terms of the redundancy analysis, the first two axes of the RDA accounted for 21.81% and 14.78% of the total variance in the data, respectively.

**FIGURE 14:**
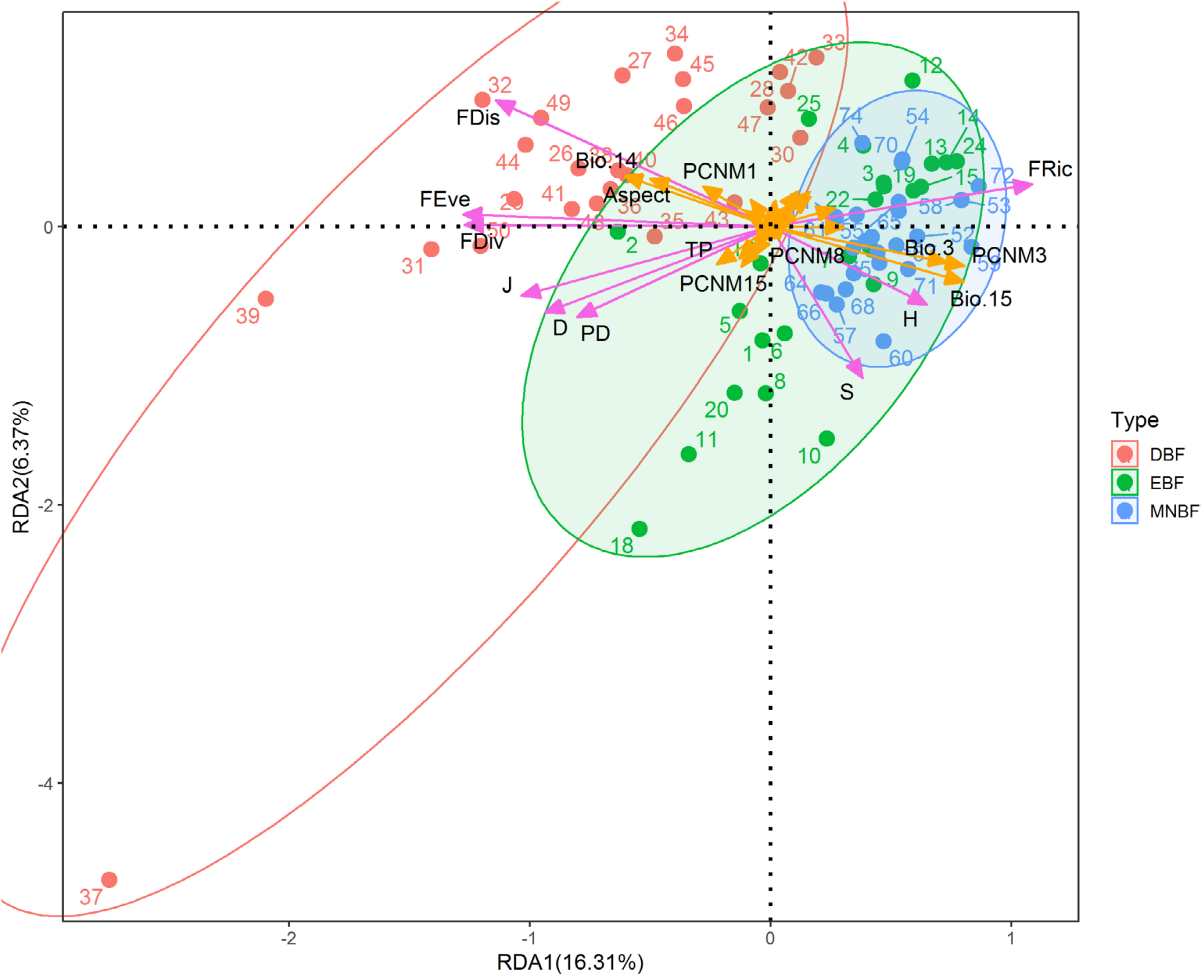
Redundancy Analysis (RDA) of the relationships among biodiversity, spatial variables, and environmental factors within the shrub canopy at Mt. Huangshan.

In the shrub layer, biodiversity was concentrated in all plots except the two plots in DBF (Figure 14). The RDA ordination diagram showed that climate factors (Bio.15, Bio.14, Bio.3) were larger correlated with biodiversity than the spatial distance, accounting for 16.31% and 6.37% of the first two axes of the RDA, respectively.

## 4 Discussion

### 4.1 Multi‐Dimensional Biodiversity Characteristics of Three Communities at mt. Huangshan

This study combined three communities at Mt. Huangshan and investigated species diversity, phylogenetic diversity, and functional diversity. The species richness of three communities was as follows: MNBF > EBF > DBF, indicating that the change of community type was accompanied by the change of species diversity (Li et al. 2024). In general, species diversity increases gradually during community succession, from the beginning to the middle of succession (Odum 1969). In this study, MNBF and EBF were in the secondary forest community of late succession, while DBF was in the secondary forest community of middle succession, so the species diversity index of MNBF and EBF was significantly higher than that of DBF. Moreover, the species diversity within the community was shaped through habitat heterogeneity. Specifically, the topographic factors of MNBF are relatively intricate. Situated at a higher elevation, it receives ample sunlight and experiences minimal human interference. These conditions facilitate the coexistence of a greater number of species. The vertical structure of species diversity for the shrub layer in each community was larger than for the tree layer, which is consistent with the results in Gutianshan Nature Reserve studied by Qian et al. (2018). This was in line with the principle that species diversity was higher in the understory of subtropical forest communities. This phenomenon might be attributed to the fact that the shrub layer harbors a greater number of tree saplings.

Phylogeny can reflect the complex adaptive strategies of species, and may be closely related to community assembly mechanisms (Baraloto et al. 2012). For EBF and DBF, phylogenetic diversity decreases with increasing elevation, which was often referred to as the expected effect of filtering in harsh high‐altitude tropical mountain environments (Gastauer et al. 2020). Surprisingly, our results indicated that the phylogenetic diversity of communities at higher elevations was higher. This finding was inconsistent with the study results of Galván‐Cisneros et al. (2023). One possible reason might be a suitable temperature, higher species richness and stand density, and closer species affinity in MNBF. In this region, where rapid speciation processes were prevalent, phylogenetic relationships among closely related species exhibit greater divergence. This pattern aligns with mountainous areas serving as major biodiversity centers, which are significantly influenced by high environmental heterogeneity and pronounced geographic isolation (Trigas et al. 2013; Noroozi et al. 2018). It was necessary for us to further enhance our comprehension of the hilltop conditions of forest communities at Mt. Huangshan.

Functional diversity reflects its ability to adapt to the environment, and is also the product of environmental screening and interspecific interaction (Islam et al. 2024). We discovered significant variation in functional diversity across different mountains. On the majority of mountains, functional richness declined with increasing elevation. However, this decrease did not always follow a linear pattern; instead, it sometimes exhibited a mid‐elevation peak (Montao‐Centellas et al. 2020). However, FRic in MNBF with the highest elevation was significantly higher than that in DBF and EBF, indicating that DBF and EBF had more resource niches than MNBF. The higher FRic was closely related to the highest species richness. This might particularly be the case for extremely rich communities in MNBF, because the chances that a new species will possess an especially unique trait set or belong to a unique phylogenetic lineage are likely low (Boris and Georgy 2025). The values of FDis, FDiv, and FEve in DBF were significantly higher than those in the other two communities, suggesting high niche differentiation, high niche overlap, and high utilization of available resources in DBF (Mouchet et al. 2010). The proportion of low‐density species in DBF made the species traits evenly distributed in space, and the effective use of resources was significantly higher than that of the other two communities. Our results highlighted the fact that the weakness of niche overlap between species might result from the competition for resources.

### 4.2 Relationship Between Biodiversity of Communities at Mt. Huangshan

While both functional and phylogenetic diversity show potential for enhancing our comprehension of community assembly, only a few studies have quantified the relationship between these two aspects of biodiversity and species richness. We discovered that species, functional, and phylogenetic diversity, rather than species, functional, and phylogenetic uniqueness, jointly demonstrated that the biodiversity of different canopy layers exhibited distinct patterns (Boris and Georgy 2025). A significant positive correlation was observed between species richness and functional richness. This indicated that an increase in the number of species within a community facilitates more efficient utilization of available resources, and this finding was in line with the results of Peco et al. (2012). Our results indicated that, in comparison with functional diversity and phylogenetic diversity, species richness contributed either equally or to a greater degree to the construction of communities. This was because species richness was shaped by assembly processes that operate on the functional and phylogenetic diversity within a community (Liu et al. 2024a; Liu et al. 2024b). If the functional trait data fail to incorporate all relevant traits, species richness might better represent true functional diversity than measures of functional diversity themselves (Díaz et al. 2015). Species diversity exhibited a positive correlation with phylogenetic diversity. This was in line with the correlation regarding the biodiversity of forest communities in Northeast China, as investigated by Gao (2020), indicating that species at Mt. Huangshan community are ecologically conservative. PD in the total and shrub layer was negatively correlated with FD, and species might continue to evolve in a combination (Kluge and Kessler 2011), that is, trait differentiation and adaptive diversification can reduce the association between FD and PD. However, the most astonishing finding was the positive association between FD and PD within the tree layer. This pattern was consistent with most of the correlations reported in the literature (Hoenle et al. 2024), because the trait conservations in our study were relatively low. In addition, our results confirmed that the relationship between FRic and PD was stronger than that between FRic and species richness, indicating that each species carried more phylogenetic information than functional trait information. However, the relationship between FEve, FDis, FDiv, and species diversity was greater than that with phylogenetic diversity, which were congruent with Pavoine et al. (2013). In identifying ecological processes, species diversity served as a superior surrogate for FD compared to PD. Some researchers believed that FD and PD indices might simply be due to changes in species diversity affecting the FD and PD values (Albassatneh et al. 2023). Our findings could also contribute to the understanding that phylogeny can be regarded as a blend of impacts from various traits evolving at different paces and could serve as a proxy for functional diversity (Paquette et al. 2015). Even though the precise relationships among species diversity, functional diversity, and phylogenetic diversity might vary across different communities, these indicators have proven to be crucial for forest conservation.

In the scientific community, there was widespread agreement that conserving biodiversity should be a top priority. However, the relationships among different aspects of biodiversity were complex (Steudel et al. 2016; Huang et al. 2020). Our findings underscored that there were differences in biodiversity among different forest communities in Mt. Huangshan, which might be related to human disturbances and climate change. Light serves as one of the most crucial driving factors for plant growth, enabling plants to acquire more abundant nutrients and growth space (Albassatneh et al. 2023). Human interference, on the other hand, can hamper the growth of certain plants, thereby causing a decline in biodiversity. Moreover, MNBF exhibits a relatively high level of species diversity, housing a large number of tree species and featuring a high density. Once the carrying capacity is surpassed, the competition for space among tree species will escalate, leading to the disappearance of some tree species and inevitably resulting in a reduction in biodiversity (Song et al. 2020). In fact, the biodiversity of different communities was also interdependent. It is essential to further broaden the research scope to elucidate the significance of biodiversity for ecological stability and community formation within the ecosystem.

### 4.3 Environmental and Spatial Variables Associated With Community Assembly

The aim of our research was to quantify the comparative significance of environmental factors on community assembly. We examined the correlation between biodiversity and the environmental and spatial conditions where the three communities were situated. Environmental factors were unexpectedly more influential on biodiversity than spatial distance, indicating that environmental filtering was the dominant assembly process. Climatic factors (Bio.15, Bio.14, Bio.3) presented the higher explained variance for biodiversity in this study. According to the hydrothermal energy hypothesis, suitable precipitation and temperature provide more ecological niches for species in the community, increase the available limited resources, and allow species near and far to coexist, thus improving biodiversity (Mi et al. 2021). This discovery suggested that precipitation (Bio.15, Bio.14) exerts a significant limiting effect on biodiversity. Additionally, a comparable limiting effect was present in semi‐arid regions, where species richness and functional diversity are affected by the combined seasonal influences of altitude, temperature, and precipitation (Palmquist et al. 2018). In temperate forest ecosystems, the distribution of species is predominantly determined by the variations in ecological niches associated with environmental factors, including topography and soil properties (Liu et al. 2021). Topography, elevation, and aspect impact the biodiversity level as they are capable of influencing light availability and the photosynthesis process (Karimi et al. 2025). Overall, the relationship between topography and biodiversity differed across various mountainous regions. This was because topography significantly constrains the vegetation's full utilization of water during its growth process (Peng et al. 2020). Soil characteristics were poor predictors of biodiversity at Mt. Huangshan, but they cannot be ignored. TP can significantly affect biodiversity, and phosphorus was involved in many important metabolic processes in plants. Forests characterized by a higher organic content are prone to exhibit higher biodiversity. Furthermore, climate engages with the moisture conditions of the soil, enhancing the photosynthesis rate, which in turn further impacts biodiversity (Frank et al. 2015). We need to study how biodiversity changes with environmental gradients in the future.

The augmentation of biodiversity results in an enhancement of ecosystem functions, including productivity and stability. These relationships might be regulated by biological interactions between nutrient levels and interspecific trait variations (Mi et al. 2021). Results at regional and global scales showed that (Chen et al. 2021; Ren et al. 2023), environmental factors directly determine the pattern of ecosystem stability, which was in line with the outcomes of this research. As climate change and anthropogenic activities are changing the environment of Mt. Huangshan at an accelerated pace in recent years, enhanced environmental monitoring is required to safeguard its biodiversity and delicate ecosystem more efficiently.

Overall, our results corroborated that the impacts of environmental variables would supersede those of spatial variables in community assembly. In addition, studies on Mt. Huangshan have found a weak effect of spatial distance on community assembly, and it was possible that there was an issue regarding the limited scale of the study area. If the size of the study area was inadequate, the influence of diffusion constraints associated with spatial distance on community construction might be readily overlooked. This conclusion demonstrates that certain unaccounted‐for aspects in community construction might be attributed to the neutral theory. Consequently, this suggests that diffusion limitations were an essential factor influencing the community aggregation (Wang et al. 2025). Collectively, these variables have resulted in the spatially heterogeneous distribution of species (Antonelli et al. 2018) and the formation of biodiversity hotspots. In fact, the species distribution pattern of Mt. Huangshan was largely driven by stochastic and deterministic processes affected together.

## 5 Conclusions

Investigating the responses of every component of biodiversity to alterations in environmental conditions is an essential prerequisite for analyzing the state of regional biological resources. We concluded that species diversity positively correlated with PD and FD (FRic) and negatively correlated with FD (FEve, FDis, and FDiv) regrades of different canopy plants, suggesting that a community with a higher species diversity exhibited greater biodiversity. Species might evolve continuously within a combination, thus decreasing the correlation between FD and PD. Our results from the correlation analysis of biodiversity and multiple factors indicated that environmental filtering might be the dominant force shaping community assembly. Additionally, spatial distance also constrains the distribution of these assemblages, which is of equal importance in driving community construction. This research deepened our comprehension that the species distribution pattern in Mt. Huangshan is shaped by both stochastic and deterministic processes. Moreover, it validated the predictions regarding the impacts of climate change, topography, and soil factors on biodiversity. Consequently, in order to safeguard the biodiversity in Mt. Huangshan, boost the stability of the community, and reinforce the functions of the ecosystem, it is essential to consider various influencing factors comprehensively and adopt more effective protection measures.

## Author Contributions

**Ting Lv:** data curation (lead), formal analysis (lead), investigation (lead), methodology (lead), writing – original draft (lead), writing – review and editing (lead). **Rong Zhao:** conceptualization (equal), formal analysis (equal), investigation (equal). **Ningjie Wang:** conceptualization (equal), data curation (equal), project administration (equal). **Lei Xie:** methodology (equal). **Shuifei Chen:** methodology (equal), writing – original draft (equal). **Hui Ding:** conceptualization (lead), data curation (lead), formal analysis (lead), funding acquisition (lead), investigation (lead). **Yanming Fang:** conceptualization (lead), data curation (lead), supervision (lead), validation (lead), visualization (lead).

## Conflicts of Interest

The authors declare no conflicts of interest.

## Data Availability

The original contributions presented in the study are included in this paper; further inquiries can be directed to the corresponding author.
